# Effects of Sacubitril-Valsartan on Clinical, Echocardiographic, and Polygraphic Parameters in Patients Affected by Heart Failure With Reduced Ejection Fraction and Sleep Apnea

**DOI:** 10.3389/fcvm.2022.861663

**Published:** 2022-04-05

**Authors:** Corrado Pelaia, Giuseppe Armentaro, Mara Volpentesta, Luana Mancuso, Sofia Miceli, Benedetto Caroleo, Maria Perticone, Raffaele Maio, Franco Arturi, Egidio Imbalzano, Francesco Andreozzi, Francesco Perticone, Giorgio Sesti, Angela Sciacqua

**Affiliations:** ^1^Department of Medical and Surgical Sciences, University Magna Græcia of Catanzaro, Catanzaro, Italy; ^2^Department of Clinical and Experimental Medicine, University of Messina, Messina, Italy; ^3^Department of Clinical and Molecular Medicine, Sapienza University of Rome, Rome, Italy

**Keywords:** heart failure, sleep apnea, sacubitril-valsartan, echocardiography, apnea-hypopnea index

## Abstract

**Background:**

Heart failure with reduced ejection fraction (HFrEF) is a clinical condition frequently diagnosed in clinical practice. In patients affected by HFrEF, sleep apnea (SA) can be detected among the most frequent comorbidities. Sacubitril–valsartan (sac/val) association has been proven to be effective in reducing disease progression and all-cause mortality in HFrEF patients. Sac/val treatment can potentially attenuate SA development *via* several pathophysiologic mechanisms, including improvement of global hemodynamics, reduction of extracellular fluid overload, and decrease of sympathetic neural activity.

**Methods:**

We recruited 132 patients affected by HFrEF and SA, already under treatment with continuous positive airway pressure (CPAP), which was discontinued 24 h before the scheduled study timepoints. Physical examination, echocardiography, nocturnal cardio-respiratory monitoring, and laboratory tests were performed in each patient at baseline and after a 6-month treatment with sac/val.

**Results:**

After 6 months, sac/val induced statistically significant changes in clinical, hemodynamic, biohumoral (NT-proBNP, serum electrolytes, creatinine, and uric acid), and echocardiographic parameters. In particular, cardiac index (CI), both atrial and ventricular volumes and global longitudinal strain (GLS) improved. Moreover, polysomnography, carried out during a temporary CPAP interruption, revealed a significant reduction in global apnea-hypopnea index (AHI) value (*p* < 0.0001), central AHI (*p* < 0.0001), obstructive AHI (*p* < 0.0001), oxygen desaturation index (ODI) (*p* < 0.0001), and percentage time of saturation below 90% (TC90) (*p* < 0.0001). The changes of CI, estimated glomerular filtration rate (eGFR), NT-proBNP, and tricuspid annular plane excursion (TAPSE) contributed to 23.6, 7.6, 7.3, and 4.8% of AHI variability, respectively, and the whole model accounted for a 43.3% of AHI variation.

**Conclusions:**

Our results suggest that treatment with sac/val is able to significantly improve the cardiorespiratory performance of patients with HFrEF and SA, integrating the positive impact of CPAP. Thus, both CPAP and sac/val therapy may synergistically contribute to lower the risks of both cardiac and pulmonary complications in HFrEF patients with SA.

## Introduction

Heart failure (HF) is a largely diffused clinical condition, being responsible for very high social and economic costs worldwide. In Western countries, about 1–2% of adult people are affected by HF, and the phenotype characterized by reduced ejection fraction (HFrEF) represents more than half of the cases (1). In spite of the relevant improvements recently achieved in pharmacological treatment of HF, prognosis remains quite poor, being characterized by high rates of hospitalization and death (2, 3). In more than 50% of patients with HF, central sleep apneas (CSA) and obstructive sleep apneas (OSA) can be detected among the most frequent comorbidities that significantly contribute to all-cause mortality (4–6). Many therapeutic strategies have been suggested to treat both CSA and OSA, but so far, no pharmacologic treatment is providing significant prognostic benefits for patients with HF (7–9). Although OSA can be regarded as an independent risk factor enhancing HF morbidity and mortality (10, 11), CSA seems to be an important indicator of HF severity, due to its association with a worse left ventricular function and an advanced New York Heart Association (NYHA) class (12). All sleep apnea (SA) phenotypes are characterized by an increased sympathetic activity, which represents a detrimental pathophysiologic condition for HF patients. In particular, an exaggerated function of the sympathetic neural pathway leads to an excessive activation of renin–angiotensin–aldosterone system (RAAS), responsible for high renal resorption of salt and water, associated with both increased heart rate (HR) and peripheral vasoconstriction (13, 14). Furthermore, hyperactivation of the adrenergic system directly causes vasoconstriction, as well as increases the overall arrhythmic risk. Therefore, SA is thought to be a potential target for therapeutic approaches which can improve the health status of HF patients (15). Whilst current evidence indicates that OSA treatment with continuous positive airway pressure (CPAP) decreases all-cause mortality in HF patients (16–18), the benefits of non-invasive ventilation (NIV) for CSA therapy are still debated (19–21), as suggested by the results of both SERVE-HF (22) and ADVENT-HF (23) randomized clinical trials.

Moreover, it is well-known that optimization of HFrEF therapy should represent the first line treatment of subjects affected by SA and HF (24, 25). In this scenario, sacubitril–valsartan (sac/val) association has been proven to be more effective than enalapril in reducing HF progression and all-cause mortality in HFrEF patients (26). This therapeutic combination inhibits neprilysin *via* LBQ657, the active metabolite of the prodrug sacubitril, and blocks the angiotensin II type-1 (AT1) receptor *via* valsartan. In a real-life setting, we have recently demonstrated that sac/val treatment is effective and safe, as shown by the long-lasting benefits including significant improvements in several clinical, hemodynamic, and echocardiographic parameters, observed in HFrEF outpatients monitored every 6 months up to 2-years (27). Indeed, sac/val is the gold-standard treatment of patients suffering from HFrEF, whose symptoms persist in spite of an optimized therapy including an angiotensin-converting enzyme inhibitor (ACE-I) or angiotensin receptor blockers (ARB), a beta-blocker, and a mineralocorticoid receptor antagonist (MRA) (26, 28, 29). Notably, sac/val treatment can potentially attenuate sleep apnea development *via* several mechanisms including improvement of global hemodynamics, decrease of extracellular fluid overload, and reduction of sympathetic neural activity (30).

To address this issue, we aimed to evaluate the effects of a 6-month therapy with sac/val on hemodynamic and metabolic parameters, as well as on apnea/hypopnea occurrence, and oxygen saturation in patients with HFrEF and SA, already under treatment with CPAP.

## Methods

### Study Design and Endpoints

The study population consisted of 132 consecutive outpatients enrolled from March 2018 to January 2020, referring to both the Chronic Heart Failure Unit of the Geriatrics Division, located at the “Mater Domini” University Hospital of Catanzaro, Italy, and the Internal Medicine Unit—Center for the Prevention, Diagnosis, and Management of Cardiovascular Disease, located at the University Hospital of Messina, Italy.

The study included outpatients complaining of HFrEF and eligible for treatment with sac/val, because of symptom persistence despite an optimized therapy. They were recruited according to the indications of the previous European Society of Cardiology (ESC) guidelines for the diagnosis and treatment of acute and chronic HF (31), which have been later updated, after the completion of our study. The eligibility criteria included: written informed consent; age ≥ 18 years; left ventricular ejection fraction (LVEF) ≤ 35%; NYHA class II–III; persistence of symptoms despite an optimized treatment with stable doses of ACE-Is or ARBs for at least 4 weeks; presence of SA under treatment with CPAP since at least 3 months. SA diagnosis was performed according to the current guidelines (32). No patient took drugs or other substances that could interfere with sleep. The exclusion criteria included: severe renal dysfunction (estimated glomerular filtrate-eGFR <30 mL/min/1.73 m^2^); severe hepatic impairment (Child-Pugh Class C); history of angioedema or side effects induced by ACE inhibitors or ARBs; pregnancy or breastfeeding, systolic blood pressure (SBP) < 100 mmHg; serum potassium levels > 5.4 mmol/L; current treatment with sac/val; chronic obstructive pulmonary disease (COPD) and relevant valvular heart diseases (VHD); resynchronization therapy within 3 months before the enrolment.

Complete physical examination, including assessments of body mass index (BMI), body surface area (BSA), waist circumference (WC), NYHA functional class, and quality of life were evaluated in each patient. Moreover, 12-lead electrocardiogram (ECG), echocardiography and laboratory tests aimed to evaluate metabolic disorders were also carried out. The evaluation of NYHA functional class was performed as suggested by the ESC guidelines for the diagnosis and treatment of acute and chronic HF (31). Minnesota Living with Heart Failure Questionnaire (MLHFQ) (33) and Epworth Sleepiness Scale (34) were used to evaluate quality of life. Moreover, arterial blood pressure (BP) was also checked. The local Ethics Committee approved the study protocol, and informed consent was obtained from all participants. All investigations were carried out accordingly to the principles of Helsinki Declaration.

In addition to previous treatments, eligible patients for sac/val discontinued ACE-I (at least 36 h before) or ARB, and received initial dosages of 24/26 mg or 49/51 mg bid according to clinical conditions. Moreover, sac/val dosage was increased every 2–4 weeks up to the maximum tolerated dose, as recommended. All clinical, laboratory and instrumental evaluations, such as echocardiography and nocturnal cardio-respiratory monitoring (CRM), were performed at baseline and after a 6-month treatment with sac/val. All patients were under treatment with CPAP, which provided a satisfactory SA correction. In order to evaluate the direct effects of sac/val on SA, at both baseline and 6-month timepoints CRM was performed during a temporary CPAP interruption, lasting 24 h.

### Polygraphic Parameters

All patients underwent nocturnal CRM, as previously described (35). In particular, our CRM device (Somtè, Compumedics, Australia) included five cables used to record electrocardiogram through two bipolar leads, a nasal cannula assessing the flow-meter trace, a microphone which was necessary to record snoring, two piezoelectric belts utilized to detect thoraco-abdominal movements, a digital pulse oximeter measuring peripheral arterial oxyhemoglobin saturation (SpO_2_), and a gravity sensor able to localize patient's position. Heart rate (HR) values were also assessed. All recorded parameters were analyzed by the same operator, who was blinded to treatment protocol, and each event was judged to be either obstructive, central and/or mixed, apnoic and/or hypopnoic, according to the criteria of the American Academy of Sleep Medicine (32). The eventual presence of SA was checked, and patient population was stratified on the basis of baseline central/obstructive apnea-hypopnea index (AHI) values. Hypopneas were characterized according to a scoring system which established the threshold of 3% oxygen desaturation (32). On the basis of the presence or absence of respiratory effort, sleep apneas were classified as either obstructive or central, respectively (32). We diagnosed OSA when AHI was ≥5 events/h, and >50% of apneic events were obstructive, whilst we made a diagnosis of CSA when AHI was ≥5 events/h, and >50% of apneic events were central (32). Oxygen desaturation index (ODI) was defined as the number of desaturation episodes exceeding the percentage of 3% per hour of sleep. Moreover, we also recorded time below 90% (TC90), corresponding to the percentage time of saturation which resulted to be <90%.

### Laboratory Parameters

All laboratory measurements were performed after at least 12 h of fasting. Blood levels of glucose, creatinine, uric acid (UA), total cholesterol, low-density lipoprotein (LDL) cholesterol, high-density lipoprotein (HDL) cholesterol, and triglycerides were measured. The estimation of glomerular filtration rate (eGFR) was based on the new CKD-EPI (Chronic Kidney Disease Epidemiology Collaboration) equation (36). The high sensitivity C-reactive protein (hs-CRP) and serum N-terminal pro-B-type natriuretic peptide (NT-proBNP) were also measured.

### Echocardiographic Parameters

Standard left ventricular ultrasonography in both M-mode (motion mode) and B-mode (two-dimensional mode) was performed in all patients, according to the recommendations of the American Society of Echocardiography (37). Recordings were made using a VIVID E-95 ultrasound system (GE Technologies, Milwaukee, Wisconsin, USA) and a 2.5 MHz transducer. Echocardiographic parameters were detected by the same expert operator, in order to minimize measurement errors. However, the operator was not aware of patient's clinical data and the values represented the average of at least three measurements. Among the parameters of left ventricular global systolic function, LVEF, cardiac output (CO), and cardiac index (CI) were evaluated. Right ventricular systolic parameters were also measured, by estimating the systolic pulmonary arterial pressure (S-PAP). Diastolic dysfunction was detected by recording pulse-wave Doppler patterns at the mitral valve, in order to measure early (E) and late (A) diastolic filling velocities from the 4-chamber view. Assessment of the left ventricular global longitudinal strain (GLS) and multilayer parameters were obtained using a dedicated software (38).

### Statistical Analysis

Continuous variables were expressed as mean ± standard deviation (SD) (normally distributed data) or as median with interquartile range (IQR) (non-normally distributed data), when appropriate. Categorical data were expressed as numbers and percentages. Normally distributed data were analyzed by *t*-test for paired data, whereas non-normally distributed data were analyzed by Wilcoxon's test for paired data. Subsequently, because of the absence of binary outcomes, a simple linear regression model was constructed with delta (**Δ**) as the dependent variable, i.e., changes in AHI between follow-up and baseline, and deltas of different variables that differed in a statistically significant manner between follow-up and baseline as independent variables. Therefore, changes in the variables that correlated significantly with changes in the dependent variable were entered into a multivariate linear regression model. Statistical analysis was carried out using SPSS V20.0 program for Windows (SPSS Inc., Chicago, Illinois, USA).

## Results

The recruited subjects included 107 men (81.1%) and 25 women (18.9%), with an average age of 67 ± 9.8 years. Table 1 shows the baseline characteristics of the entire study population. In details, 59 (44.7%) patients received a treatment with sac/val 24/26 mg, 45 (34.1%) subjects assumed a dose of 49/51 mg, and 28 (21.2%) individuals received a 97/103 mg dose. Moreover, 66 (50.0%) patients had an implantable cardioverter defibrillator (ICD), and 42 (31.8%) subjects had a cardiac resynchronization therapy (CRT). After a 6-months follow-up, 39 (29.5%) patients were taking the lowest dose of sac/val (24/26), 60 (45.5%) patients the intermediate dose (49/51), and 33 (25%) the highest dose (97/103). Comparative evaluation between baseline values and 6-months follow-up data showed significant improvements in clinical and hemodynamic parameters, including reductions in HR (76.2 ± 2.8 vs. 71.7 ± 7.5 bpm; *p* < 0.0001), respiratory rate (RR) (17.7 ± 2.8 vs. 16.0 ± 1.8 breaths/min; *p* < 0.0001), SBP (122.1 ± 11.7 vs. 119.1 ± 11.7 mmHg; *p* < 0.0001), diastolic blood pressure (DBP) (72.4 ± 7.6 vs. 69.9 ± 6.7 mmHg; *p* < 0.0001). Furthermore, there was a significant change in MLHFQ score (89.5 ± 3.4 vs. 84.4 ± 4.5; *p* < 0.0001), indicating a relevant improvement in clinical symptoms (Table 2). After 6 months of treatment with sac/val, we also noticed significant improvements in patient functional status, as shown by the significant reduction of individual with NYHA class III from 68.9 to 22.0%; *p* < 0.0001.

**Table 1:** Baseline characteristics and comorbidities of enrolled patients.

**Variables**	**Baseline**
	**(*n* = 132)**
Age, years	67.0 ± 9.8
Gender, m/f	107/25
Ischemic heart disease, *n* (%)	96 (72.7)
Diabetes, *n* (%)	80 (60.6)
Atrial fibrillation, *n* (%)	45 (34.0)
Arterial hypertension, *n* (%)	114 (86.3)
Dyslipidemia, *n* (%)	112 (84.8)
NYHA functional class II, *n* (%)	41 (31.1)
NYHA functional class III, *n* (%)	91 (68.9)
Smokers, *n* (%)	28 (21.2)
Ex-smokers, *n* (%)	24 (18.2)
Hemoglobin, g/dl	12.0 ± 1.2
Implantable cardioverter defibrillator, *n* (%)	66 (50.0)
Cardiac resynchronization therapy, *n* (%)	42 (31.8)

*NYHA, New York Heart Association*.

**Table 2:** Anthropometric, hemodynamic, and biohumoral characteristics at baseline and after 6 months of therapy with sac/val.

**Variables**	**Baseline**	**Follow-up**	**Standardized mean difference**	***p***
	**(*n* = 132)**	**(*n* = 132)**	**(Hedges'g)**	
BMI, kg/m^2^	32.0 ± 4.6	31.1 ± 4.4	0.19	<0.0001
SBP, mmHg	122.1 ± 11.7	119.1 ± 11.7	0.25	<0.0001
DBP, mmHg	72.4 ± 7.6	69.9 ± 6.7	0.35	<0.0001
HR, bpm	76.2 ± 2.8	71.7 ± 7.5	0.79	<0.0001
RR, breaths/min	17.7 ± 2.8	16.0 ± 1.8	0.72	<0.0001
MLHFQ, score	89.5 ± 3.4	84.4 ± 4.5	1.27	<0.0001
Serum sodium, mmol/L	140.4 ± 2.1	139.7 ± 1.6	0.37	<0.0001
Serum potassium, mmol/L	4.4 ± 0.3	4.6 ± 0.3	0.67	<0.0001
Serum creatinine, mg/dL	1.1 ± 0.3	1.0 ± 0.2	0.39	<0.0001
eGFR, mL/min/1.73m^2^	67.2 ± 19.2	96.4 ± 31.0	1.13	<0.0001
Serum UA, mg/dL	6.7 ± 0.8	5.9 ± 1.0	0.88	<0.0001
hs-CRP, mg/dL	7.4 ± 0.4	6.6 ± 0.4	2	<0.0001
NT-proBNP, pg/mL	1840 (886.0–3,378)	970.0 (571.3–2,870)		<0.0001

*BMI, body mass index; SBP, systolic blood pressure; DBP, diastolic blood pressure; HR, heart rate; RR, respiratory rate; MLHFQ, Minnesota Living with Heart Failure Questionnaire; eGFR, estimated glomerular filtration rate; UA, uric acid; hs-CRP, high-sensitivity C-reactive protein; NT-proBNP, N-terminal pro-B-type natriuretic peptide*.

There were also statistically significant decreases of BMI (32.9 ± 4.6 vs. 31.1 ± 4.4 kg/m^2^; *p* < 0.0001), hs-CRP (7.4 ± 0.4 vs. 6.6 ± 0.4 mg/L; *p* < 0.0001), serum uric acid (6.7 ± 0.8 vs. 5.9 ± 1.0 mg/dL; *p* < 0.0001), NT-proBNP levels [1,840 (886.0–3,378] pg/mL vs. 970.0 (571.3–2,870) pg/mL; *p* < 0.0001). Furthermore, there was a significant reduction of serum creatinine levels (1.1 ± 0.3 vs. 1.0 ± 0.2 mg/dL; *p* < 0.0001), associated with a significant increase in eGFR (67.2 ± 19.2 vs. 96.4 ± 31.0 mL/min/1.73 m^2^; *p* < 0.0001). Additionally, a significant reduction in sodium (140.4 ± 2.1 vs. 139.7 ± 1.6 mmol/L; *p* < 0.0001) and potassium (4.4 ± 0.3 vs. 4.6 ± 0.3 mmol/L; *p* < 0.0001) serum levels were observed (Table 2).

Table 3 shows the echocardiographic characteristics of the study group. The echocardiographic analysis revealed an improvement of both left chambers diameters, as demonstrated by the reduction of LAVI, from 49.8 ± 13.7 to 46.1 ± 12.0 mL/m^2^ (*p* = 0.001). Moreover, LVEDV/BSA decreased from 89.6 ± 9.8 to 87.8 ± 8.4 mL/m^2^ (*p* < 0.0001), and LVESV/BSA from 61.0 ± 7.1 to 57.3 ± 5.9 mL/m^2^ (*p* < 0.0001), respectively. We also found significant reductions of the right chambers diameters, as shown by the changes in right ventricular outflow tract (RVOT), which decreased from 2.6 ± 0.4 to 2.1 ± 0.4 cm (*p* < 0.0001), and in the area of the right atrium (RAA) which decreased from 20.5 ± 2.8 to 19.3 ± 2.3 cm^2^ (*p* < 0.0001). A statistically significant reduction was found in left ventricular GLS, changing from −7.9 ± 1.7 to −9.0 ± 1.4% (*p* < 0.0001). Moreover, s-PAP changed from 44.5 ± 6.6 to 41.5 ± 6.6 mmHg (*p* < 0.0001) and E/e' decreased from 17.4 ± 3.5 to 15.9 ± 2.8 (*p* < 0.0001). In addition, we detected statistically significant improvement in LVEF, which increased from 31.9 ± 1.4 to 34.7 ± 1.6% (*p* < 0.0001), as well as in CI, which enhanced from 1,675.6 ± 199.9 to 1,856.6 ± 212.9 mL/min/m^2^ (*p* < 0.0001); inferior vena cava (IVC) diameter decreased from 20.2 ± 1.3 to 19.1 ± 3.3 mm (*p* < 0.0001). Furthermore, we observed a statistically significant increase in tricuspid annular plane excursion (TAPSE), which enhanced from 16.3 ± 1.1 to 17.1 ± 1.7 mm (*p* < 0.0001). The TAPSE/S-PAP ratio improved from 0.37 ± 0.06 to 0.42 ± 0.08 mm/mmHg (*p* < 0.0001).

**Table 3:** Echocardiographic parameters at baseline and after 6 months of therapy with sac/val.

**Variables**	**Baseline**	**Follow-up**	**Standardized mean difference**	***p***
	**(*n* = 132)**	**(*n* = 132)**	**(Hedges'g)**	
LAVI, mL/m^2^	49.8 ± 13.7	46.1 ± 12.0	0.27	0.001
LVEDV/BSA, mL/m^2^	89.6 ± 9.8	87.8 ± 8.4	0.20	<0.0001
LVESV/BSA, mL/m^2^	61.0 ± 7.1	57.3 ± 5.9	0.57	<0.0001
LVEF, %	31.9 ± 1.4	34.7 ± 1.6	1.86	<0.0001
CI, mL/min/m^2^	1,675.6 ± 199.9	1,856.6 ± 212.9	0.87	<0.0001
E/e' ratio	17.4 ± 3.5	15.9 ± 2.8	0.47	<0.0001
GLS, %	−7.9 ± 1.7	−9.0 ± 1.4	0.71	<0.0001
RVOT, cm	2.6 ± 0.4	2.1 ± 0.4	1.25	<0.0001
RAA, cm^2^	20.5 ± 2.8	19.3 ± 2.3	0.4	<0.0001
TAPSE, mm	16.3 ± 1.1	17.1 ± 1.7	0.56	<0.0001
S-PAP, mmHg	44.5 ± 6.6	41.5 ± 6.6	0.45	<0.0001
TAPSE/S-PAP, mm/mmHg	0.37 ± 0.06	0.42 ± 0.08	0.70	<0.0001
IVC, mm	20.2 ± 1.3	19.1 ± 3.3	0.44	<0.0001

*LAVI, left atrium volume index; LVEDV, left ventricular end-diastolic volume; BSA, body surface area; LVESV, left ventricular end-systolic volume; LVEF, left ventricular ejection fraction; CI, cardiac index; GLS, left ventricular global longitudinal strain; RVOT, right ventricular outflow tract; RAA, right atrium area; TAPSE, tricuspid annular plane excursion; S-PAP, systolic pulmonary arterial pressure; IVC, inferior vena cava*.

**Figure 1:**
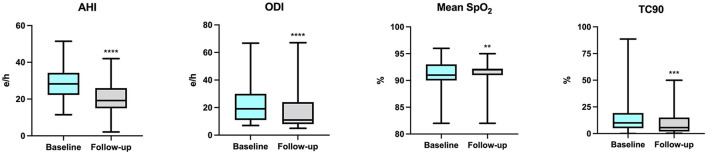
Changes of AHI, ODI, mean SpO_2_ and TC90 after 6 months of therapy with sac/val in the subgroup with OSA. AHI, apnea hypopnea index; ODI, oxygen desaturation index; SpO_2_, peripheral arterial oxyhemoglobin saturation; TC90, percentage time of saturation below 90%; OSA, obstructive sleep apnea. **p < 0.01; ***p < 0.001; ****p < 0.0001.

**Figure 2:**
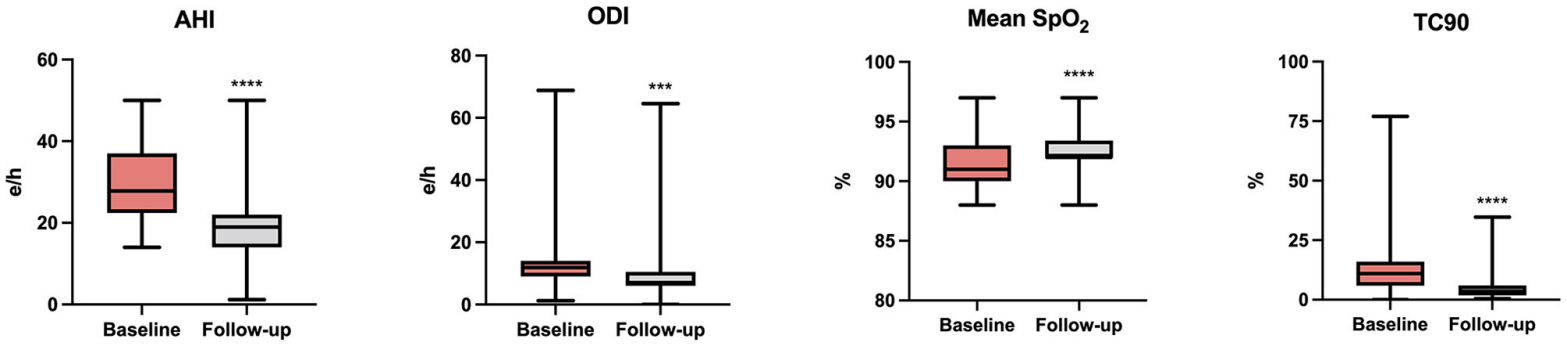
Changes of AHI, ODI, mean SpO_2_, and TC90 after 6 months of therapy with sac/val in the subgroup with CSA. AHI, apnea hypopnea index; ODI, oxygen desaturation index; SpO_2_, peripheral arterial oxyhemoglobin saturation; TC90, percentage time of saturation below 90%; CSA, central sleep apnea. ***p < 0.001; ****p < 0.0001.

At baseline, OSA and CSA were diagnosed in 55 (41.7%) and 77 (58.3%) patients, respectively, and all patients were under treatment with CPAP. Assessment of polygraphic parameters, evaluated during a temporary CPAP interruption, revealed a significant reduction in global AHI value (26.5 ± 10.4 vs. 21.7 ± 8.3 e/h; *p* < 0.0001), ODI (18.0 ± 3.7 vs. 13.5 ± 4.9 e/h; *p* < 0.0001), and TC90 (14.1 ± 4.5 vs. 6.8 ± 3.9%; *p* < 0.0001), respectively. Moreover, significant elevations were detected in mean SpO_2_, which increased from 91.3 ± 1.9 to 92.0 ± 2.0% (*p* < 0.0001) (Table 4). In addition, apnea severity improved, as indicated by the reduction of patients with severe apneas from 58 (43.9%) at baseline to 15 (11.4%) after 6 months of therapy with sac/val (*p* < 0.0001). Significant improvements of AHI, ODI, mean SpO_2_ and TC90 were also observed in both subgroups of patients with OSA (Figure 1) or CSA (Figure 2). The mean value of CPAP use time was 6.3 ± 0.17 h/night, remaining stable over time (6.3 ± 0.19 h/night, *p* = 0.999) thus reflecting the good patient compliance to device utilization.

**Table 4:** Polygraphic parameters at baseline and after 6 months of therapy with sac/val.

**Variables**	**Baseline**	**Follow-up**	**Standardized mean difference**	***p***
	**(*n* = 132)**	**(*n* = 132)**	**(Hedges'g)**	
AHI, e/h	26.5 ± 10.4	21.7 ± 8.3	0.51	<0.0001
ODI, e/h	18.0 ± 3.7	13.5 ± 4.9	1.03	<0.0001
Mean SpO_2_, %	91.3 ± 1.9	92.0 ± 2.0	0.36	<0.0001
TC90, %	14.1 ± 4.5	6.8 ± 3.9	1.73	<0.0001

*AHI, apnea hypopnea index; ODI, oxygen desaturation index; SpO_2_, peripheral arterial oxyhemoglobin saturation; TC90, percentage time of saturation below 90%*.

Table 5 shows the differences occurring between the pharmacological treatments received at baseline and after 6 months of sac/val therapy, respectively. Notably, there was a significant reduction in diuretic drugs use.

**Table 5:** Pharmacological treatments at baseline and after 6 months of therapy with sac/val.

**Variables**	**Baseline**	**Follow-up**	***p***
	**(*n* = 132)**	**(*n* = 132)**	
ACE-I, *n* (%)	92 (69.7)	0 (0)	<0.0001
ARB, *n* (%)	40 (30.3)	0 (0)	<0.0001
MRA, *n* (%)	68 (51.5)	49 (37.1)	0.018
Loop diuretics, *n* (%)	131 (99.2)	115 (87.1)	<0.0001
Beta blockers, *n* (%)	131 (99.2)	131 (99.2)	0.999
Antiplatelet agents, *n* (%)	72 (54.5)	72 (54.5)	0.999
Oral antidiabetic drugs, *n* (%)	80 (60.6)	73 (55.3)	0.382
Oral anticoagulants, *n* (%)	44 (33.3)	44 (33.3)	0.999
Statins, *n* (%)	109 (82.6)	109 (82.6)	0.999

*ACE-I, angiotensin-converting enzyme inhibitor; ARB, angiotensin receptor blockers; MRA, mineralocorticoid receptor antagonist*.

Linear regression analysis using AHI variation (Δ AHI) as dependent variable revealed that AHI changes were significantly associated with variations of eGFR, CI, NT-proBNP, and TAPSE (Table 6). Moreover, variables significantly correlated to AHI changes were inserted in a multivariate linear regression model to determine the independent predictors of AHI changes. We found that the changes of CI, eGFR, NT-proBNP, and TAPSE contributed to 23.6, 7.6, 7.3, and 4.8% of AHI variability, respectively, and the whole model accounted for a 43.3% of AHI variation (Table 7).

**Table 6:** Linear regression analysis focused on AHI variation (Δ AHI) as dependent variable.

**Variables**	***R***	***p***
Δ CI, mL/min/m^2^	0.486	<0.0001
Δ TAPSE, mm	0.325	<0.0001
Δ NT-proBNP, pg/mL	−0.404	<0.0001
Δ IVC, mm	−0.048	0.293
Δ eGFR, mL/min/1.73m^2^	0.405	<0.0001
Δ RAA, cm^2^	−0.086	0.164
Δ MLHFQ, score	−0.066	0.228
Δ RVOT, cm	−0.104	0.218
Δ TAPSE/S-PAP, mm/mmHg	0.036	0.340
Δ E/e' ratio	−0.008	0.463
Δ LVEDV/BSA, mL/m^2^	−0.022	0.401
Δ S-PAP, mmHg	−0.055	0.264

*AHI, apnea hypopnea index; CI, cardiac index; TAPSE, tricuspid annular plane excursion; NT-proBNP, N-terminal pro-B-type natriuretic peptide; IVC, inferior vena cava; eGFR, estimated glomerular filtration rate; RAA, right atrium area; MLHFQ; Minnesota Living with Heart Failure Questionnaire; RVOT, right ventricular outflow tract; S-PAP, systolic pulmonary arterial pressure; LVEDV, left ventricular end-diastolic volume; BSA, body surface area*.

**Table 7:** Stepwise multiple regression analysis focused on AHI variation (Δ AHI) as dependent variable.

**Variables**	**Partial *r*^2^ (%)**	**Total *r*^2^ (%)**	***p***
Δ CI, mL/min/m^2^	23.6	23.6	<0.0001
Δ eGFR, mL/min/1.73 m^2^	7.6	31.2	<0.0001
Δ NT-proBNP, pg/mL	7.3	38.5	<0.0001
Δ TAPSE, mm	4.8	43.3	0.001

*AHI, apnea hypopnea index; CI, cardiac index; eGFR, estimated glomerular filtration rate; NT-proBNP, N-terminal pro-B-type natriuretic peptide; TAPSE, tricuspid annular plane excursion*.

Therapy with sac/val was well-tolerated, and no serious adverse reactions occurred throughout this real-life observational study.

## Discussion

The purpose of the present observational study was to evaluate the effects of sac/val on AHI, as well as on other clinical, functional and bio-humoral parameters patients affected by HFrEF and SA syndrome, under treatment with CPAP. In this regard, the ENTRESTO-SAS study has already shown that treatment with sac/val of patients with HFrEF and respiratory sleep disorders, induced significant AHI improvements after 3 months of therapy (39). Differently from the ENTRESTO-SAS study, our investigation was extended to 6 months, and was based not only on AHI evaluation, but also on polysomnography, as well as on the assessment of several other clinical, echocardiographic, and laboratory parameters. We observed significant improvements of AHI, ODI, TC90, mean SpO_2_, LVEF, CI, S-PAP, MLHFQ score, NT-proBNP, and eGFR in patients treated with sac/val for 6 months. Because all enrolled patients were on treatment with CPAP throughout the entire study period with a good adherence to non-invasive ventilation, the observed effects were likely to be attributable to the pharmacological action of sac/val. Indeed, this drug association combines angiotensin receptor antagonism and neprilysin inhibition. As a consequence of this dual mechanism of action, the resulting effective vasodilation can lead to a decrease of peripheral, and pulmonary congestion (40–43). In fact, vasodilation arises from suppression of angiotensin-dependent vasomotor tone, as well as from inhibition of degradation of vasodilatory natriuretic peptides (NP). This powerful vasodilation is further potentiated by natriuresis, ensuing from NP increased levels, and also from RAAS inhibition. The natriuretic action of sac/val also contributes to enhance eGFR and to lower serum UA levels, which are well-known cardiovascular risk factors (44, 45). Furthermore, the improved renal function decreases pulmonary congestion, thus explaining why eGFR change is a significant contributor to AHI variation in the multivariate linear regression model. Taken together, these therapeutic effects amplify a virtuous feed-forward loop, leading to LVEF and CI increases, as well as to partial reversal of cardiac remodeling (46, 47). Additionally, sac/val treatment leads to reduction in water content of lung interstitial spaces, thereby improving pulmonary congestion, ventilation dynamics, and gas exchange (48). Overall, these effects are also responsible for a decreased stimulation of lung stretch receptors, which positively impacts on respiratory mechanics (49). The increased cardiac output is paralleled by a decreased circulation time, associated with a reduced sensitiveness of the chemoreceptor reflex system to blood gas changes (50). Within the context of SA pathophysiology, the above pulmonary and cardiovascular improvements can contribute to dampen the hyperactivation of sympathetic neural pathways (51). Furthermore, sac/val treatment could reduce the cranial fluid influx occurring when patients like ours rest in supine position (52).

Similar to the characteristics of the PARADIGM-HF trial (26), also our patients experienced an overall very good response to sac/val. Moreover, our results are consistent with those of a very recent study carried out by Passino et al. (53), who showed that sac/val combination significantly improved the apnea burden due to CSA in patients with HFrEF; however, these authors did not detect significant changes in nighttime obstructive AHI referred to OSA patients. By contrast, we observed significant improvements in AHI which regarded both CSA and OSA in a quite larger number of enrolled patients. This discrepancy could be explained by the different baseline features of our patients, when compared with those enrolled by Passino et al., especially with regard to TC90 and AHI values. Additionally, despite the interruption of CPAP during the night of recording, some residual effects of mask treatment make explain the positive effects on OSA. Anyway, the two subgroups of patients (OSA and CSA), recruited in our study, do not appear to be characterized by relevant differences with regard to both body weight and BMI. In the AWAKE-HF study, the addition of sac/val therapy did not significantly improve sleep-disordered breathing or sleep duration in a cohort of patients with HFrEF, likely as a result of the short duration of the study (54). Overall, our patients responded quite well to sac/val in terms of SA improvement. However, 96 (72.7%) patients experienced the persistence of a residual AHI higher than 15, and 12 (9.1%) subjects were characterized by a residual AHI higher than 30. In particular, the persistence of about 20 events/h in both patient subgroups (OSA and CSA) suggests that the addition of further treatments should be tested, especially in regard to subjects with high loop gain OSA (e.g., acetazolamide and oxygen) (55). Acetazolamide, buspirone, and phrenic nerve stimulation could be useful for add-on treatment of CSA (56–60).

In conclusion, our results suggest that treatment with sac/val is able to significantly improve the cardiorespiratory performance of patients with HFrEF and SA. These findings are consistent with the recommendations of the current guidelines for the diagnosis and treatment of acute and chronic HF, that consider sac/val as a first-choice treatment in HFrEF (1). This real-life observation can be clinically relevant because cardiovascular diseases represent the main comorbidities of SA patients, negatively affecting both quality of life and survival. However, our study also presents some limitations. Firstly, similarly to all real-life experiences, the enrolled patients were not randomized, and, therefore, bias selection cannot be excluded. Secondly, we used CRM instead of polysomnography equipped with electroencephalography channels, which may provide a more detailed characterization of sleep patterns. A further limitation arises from the night-to-night AHI variability, which can at least in part confound the effects of sac/val (61). Moreover, although some patients can experience central apneas even at daytime, while awake and in the upright position (62, 63), we chose to focus our attention only on nighttime registration, with the aim of increasing patient adherence. A further limitation refers to a possible misclassification of hypopneas; indeed, some residual respiratory efforts can be present even in central hypopneas. Nevertheless, the present results indicate that in patients with HFrEF and SA pharmacological treatment with sac/val can represent a valuable therapeutic option, integrating the positive impact of CPAP. Thus, both CPAP and sac/val therapy may synergistically contribute to improve the global performance of these patients, as well as to significantly lower the risks of both cardiac and pulmonary complications. Of course, it cannot be ruled out that alternative pharmacologic strategies to sac/val could be useful in different patients, characterized by heart failure with either preserved ejection fraction (HFpEF) or midrange ejection fraction (HFmrEF).

## Data Availability Statement

The raw data supporting the conclusions of this article will be made available by the authors, without undue reservation.

## Ethics Statement

The studies involving human participants were reviewed and approved by Local Ethical Committee of Calabria Region. The patients/participants provided their written informed consent to participate in this study.

## Author Contributions

All authors listed have made a substantial, direct, and intellectual contribution to the work and approved it for publication.

## Conflict of Interest

The authors declare that the research was conducted in the absence of any commercial or financial relationships that could be construed as a potential conflict of interest.

## Publisher's Note

All claims expressed in this article are solely those of the authors and do not necessarily represent those of their affiliated organizations, or those of the publisher, the editors and the reviewers. Any product that may be evaluated in this article, or claim that may be made by its manufacturer, is not guaranteed or endorsed by the publisher.
